# Genetic merit of sires for *ad libitum* residual feed intake affects feed efficiency of restricted-fed heavy pigs but not body weight gain tissue composition

**DOI:** 10.1371/journal.pone.0312307

**Published:** 2024-10-17

**Authors:** Chiara Mondin, Sara Faggion, Diana Giannuzzi, Luigi Gallo, Stefano Schiavon, Paolo Carnier, Valentina Bonfatti

**Affiliations:** 1 Department of Comparative Biomedicine and Food Science, University of Padova, Legnaro (Padova), Italy; 2 Department of Agronomy, Food, Natural Resource, Animals and Environment, University of Padova, Legnaro (Padova), Italy; University of Ghana, GHANA

## Abstract

The study aimed at evaluating how sires, classified for their additive genetic effects on residual feed intake (RFI) of *ad libitum*-fed progeny, influence growth performance, tissue accretion, and gain composition in restricted-fed offspring (96–168 kg body weight, BW). A total of 416 purebred C21 Goland pigs, offspring of 23 sires, were randomly allocated to three feeding groups: *ad libitum*, restricted medium-protein, or restricted low-protein. Empty BW, body lipid mass and body protein mass were estimated from individual BW and backfat measures using literature equations. Residuals of a linear regression of average daily feed intake on average empty BW, body lipid and protein daily gains were used as estimates of individual RFI in *ad libitum*-fed pigs. Additive genetic effects of sires on RFI of *ad libitum*-fed pigs were estimated with a linear animal model and solutions of the model were used to allocate the sires to low- (LRFI), medium- (MRFI), or high-RFI (HRFI) groups. Restricted-fed progeny of LRFI sires exhibited reduced average daily feed intake (-3%) compared to MRFI and HRFI progeny. This indicates that LRFI progeny make a more efficient use of energy intake and implies that variation in RFI across families, assessed under *ad libitum* feeding, is related to the across-family variation in feed efficiency detected under restricted feeding. LRFI progeny exhibited also a lower feed conversion ratio (-11%), partially resulting from of a 3% increase in growth rate compared with HRFI. Thus, LRFI progeny consumed less feed, while growing at a similar or slightly higher rate than MRFI or HRFI. No significant differences across sire classes were observed for daily tissue accretion and gain composition. Hence, we can hypothesise that efficient sires would not affect carcass leanness of heavy pig progeny fed restricted.

## Introduction

One of the crucial goals in pig production is maximizing feed efficiency, as it is associated with reduced environmental impact and increased profit for farmers [1–4]. Residual feed intake (**RFI**) is the difference between an animal’s observed feed intake and the expected intake based on its maintenance requirements and growth composition [5]. RFI is more accurate than other measures of feed efficiency [4] as it reflects individual differences in maintenance requirements and it is influenced by digestive and metabolic efficiency, thermoregulation, protein turnover, coping strategies to social stress, activity, feeding behaviour, and body composition [6]. In addition, RFI is characterized by a moderate heritability (0.10–0.50) [4,7]. For these reasons, selecting pig lines for low RFI is an effective strategy to increase feed efficiency [2,8].

Selection experiments aiming to establish low RFI pig lines and to investigate variation in their production and carcass traits have been conducted over the years. In 2001, two selection lines, a low RFI line and a randomly-selected control line, were established at Iowa State University [8]. After a few years, a second selection experiment involving divergent lines (i.e., low and high RFI) was conducted at INRA [9]. Overall, these studies evidenced that, after four generations of selection, animals selected for low RFI exhibited a remarkably lower RFI (from -165 to -240 g/day), a significantly reduced feed intake (from -270 to -380 g/day) and a similar or lower daily gain (from -8 to -79 g/day) [2,10], with carcasses that had [8,11,12] or tended to have less backfat (**BF**) [13,14] compared to the other lines.

Since the majority of pigs worldwide are slaughtered at 100–120 kg body weight (**BW**), research studies have focused on the relationship between RFI and production traits within a weight range of 30 to 115 kg. However, growth and maintenance requirements change over time, resulting in variation in the protein-to-fat deposition rates [15]. For this reason, results obtained in those studies might not apply to pigs in later growth stages. In addition, quantification of RFI requires pigs to be under *ad libitum* feeding conditions to enable expression of individual variation in voluntary feed intake [2,8,10] and there is limited evidence on how variation in RFI affects growth performance and carcass composition of restricted-fed pigs. Most importantly, the effects of such variation on pigs of heavy body weight are currently unknown.

Exploring the relationship between RFI and production traits at later growth stages in restricted-fed pigs is crucial for Italian pig farming, which relies predominantly on heavy pigs slaughtered at ≥ 9 months of age and 134–205 kg BW intended for the production of Protected Designation of Origin dry-cured hams. In addition to targets for age and weight at slaughter, met by carefully managing the animals growth rate through restricted feeding [16,17], product specifications require a carcass lean meat content ranging from 40% to 55% and set minimum thresholds for ham fat thickness and maximum values for iodine number and linoleic acid content in fat [18]. Moreover, the breeding programs of the pig lines approved for the production of Protected Designation of Origin dry-cured hams must be designed in order to maintain or increase the deposition of adipose tissue [19]. The potential reduction in fat deposition resulting from enhanced feed efficiency represents a limitation to the inclusion of feed efficiency traits in the breeding goal for those lines. Exploring how genetic merit of sires for *ad libitum* RFI relates to growth and body composition of restricted-fed heavy pigs may elucidate the potential outcomes resulting from the incorporation of RFI into breeding programs. Hence, the aim of the study was to evaluate how sires, classified by their estimated additive genetic effect on RFI of *ad libitum*-fed offspring, influenced growth performance, tissue accretion, and gain composition of restricted-fed progeny.

## Materials and methods

### Source of data

The experimental procedures were approved by the institutional animal care committee of the University of Padova. All procedures were conducted in compliance with the European Union requirements and guidelines on the protection of animals used for scientific and educational purposes provided by the “Organismo preposto per il Benessere Animale, OPBA”, University of Padova, approval document #36/2018 [20]. Written informed consent was obtained from the owner to allow the animals to be involved in the research.

The dataset included a total of 416 purebred C21 Goland (Gorzagri, Fonzaso, Italy) pigs (203 gilts and 213 barrows) from 4 consecutive rearing batches. Pigs were offspring of 23 sires. The number of offspring per sire was on average 18 ± 9, and ranged from 6 to 48. All the pigs were raised on the same farm, and fed the same commercial diets until their transfer to the experimental station of the University of Padova (Legnaro, Italy) at 93.6 ± 8.8 kg BW and 148 ± 1 days of age. The number of pigs in each batch ranged from 97 to 111. The 4 batches of pigs entered the experimental station sequentially from June 2018 to July 2020.

In each of the four batches, the pigs were randomly allocated to three feeding groups: *ad libitum*, fed on a high-protein diet with no limitations on indispensable amino-acid content (n = 205 in total, 4 pens per batch), restricted medium-protein (n = 104 in total, 2 pens per batch, **MP**), and restricted low-protein (n = 107 in total, 2 pens per batch, **LP**), following a split-plot design. Each sire had offspring in each of the three feeding groups. Pens were 5.8 × 3.8 m in size and the rearing density was 1.57 m^2^/pig, corresponding to 12–15 pigs per pen. Each pen was equipped with a single-space electronic feeder (Compident Pig–MLP, Schauer Agrotronic, Prambachkirchen, Austria), to record individual feed intake.

The *ad libitum* group aimed at providing progeny data to estimate additive genetic effects of the sires on RFI which were later used to classify the sires for residual feed efficiency. The MP treatment followed the conventional Italian heavy pig farming system, where pigs are slaughtered at 9 months of age and 170 kg BW. The LP treatment had the same slaughter weight target (170 kg BW), but an expected age at slaughter greater than 9 months due to the shortage of dietary protein and ileal digestible lysine. The rationale behind the use of medium or low-protein diets is related to the need to decrease feeding costs, reduce environmental impacts by lowering nitrogen emissions, and promote animal fat tissue deposition. The data from MP and LP groups were used to investigate the effects of sire classification for RFI on restricted-fed pigs.

The ingredients and the nutrient content of the diets are described in Table 1. The initial 6 days at the station were considered the adaptation of the animals to the experimental conditions and were not used in the study.

**Table 1:** Ingredient composition and nutrient content of diets.

Diet Composition^b^	Feeding group^a^					
	*Ad libitum*		Restricted			
			MP		LP	
	≤ 120 kg BW	> 120 kg BW	≤ 120 kg BW	> 120 kg BW	≤ 120 kg BW	> 120 kg BW
DM, g/kg as fed	906	906	904	902	904	904
Ingredient Composition (% on DM)						
Corn grain	35.09	38.87	34.20	39.02	38.17	39.11
Wheat grain	24.95	24.84	28.20	24.89	27.24	24.93
Barley grain	9.64	9.69	9.70	9.73	9.72	9.74
Soybean meal 48% (solv. ex.)	20.10	14.73	8.70	5.79	3.93	1.89
Wheat bran	2.55	0.72	8.44	5.58	8.26	6.07
Wheat middlings	-	3.91	1.96	6.63	2.94	8.85
Cane molasses	1.60	1.81	1.61	1.82	1.63	1.82
Lard	2.21	2.22	2.37	2.23	2.34	2.23
Dried-sugar beet pulp	-	-	0.99	0.99	1.98	2.04
Calcium carbonate	1.66	1.44	1.66	1.45	1.67	1.45
Dicalcium phosphate	0.48	0.22	0.48	0.22	0.49	0.22
Sodium chloride	0.33	0.33	0.33	0.33	0.33	0.33
Sodium bicarbonate	0.27	0.28	0.28	0.28	0.28	0.28
Vitamin and mineral premix	0.20	0.20	0.20	0.20	0.20	0.20
Grapeseed meal	0.73	0.74	0.74	0.74	0.74	0.74
Choline, liquid, 75%	0.06	-	-	-	-	-
L-Lysine	0.11	-	0.15	0.11	0.07	0.11
DL-Methionine	0.02	-	-	-	-	-
Nutrient Content						
Metabolizable energy (MJ/kg DM)	14.80	14.80	14.60	14.60	14.60	14.50
Net energy (MJ/kg DM)	11.00	11.10	11.10	11.10	11.20	11.00
Crude protein (% on DM)	17.88	15.23	14.16	13.19	12.50	11.5
Lysine (% on DM)	0.96	0.75	0.73	0.55	0.52	0.40
Methionine (% on DM)	0.30	0.28	0.24	0.22	0.21	0.20
Threonine (% on DM)	0.72	0.55	0.50	0.48	0.48	0.39
Tryptophan (% on DM)	0.20	0.14	0.17	0.12	0.13	0.11
Tyrosine (% on DM)	0.61	0.38	0.42	0.37	0.38	0.29
Fatty acid profile						
16:0 (% on DM)	8.06	8.10	8.85	8.54	8.85	8.63
18:0 (% on DM)	3.09	3.09	3.32	3.10	3.21	3.10
c18:1 (% on DM)	13.47	13.58	14.38	13.97	14.38	13.94
c18:2n-6 (% on DM)	12.58	12.91	14.05	14.08	14.49	14.38
c18:3n-3 (% on DM)	0.77	0.77	0.88	0.89	0.88	0.88

Description of diet for the early (≤ 120 kg body weight, BW) and late (> 120 kg BW) finisher period.

^a^ MP: Medium-protein diet; LP: Low-protein diet.

^b^ Vitamin and mineral premix: Providing per kilogram of feed; vitamin A, 8000 IU; vitamin D3, 1200 IU; vitamin E, 8 mg; vitamin B7, 0.08 mg; vitamin B12, 0.012 mg; niacin, 16.0 mg; biotin, 8 mg; iron, 170 mg; zinc, 117 mg; copper, 14 mg; cobalt, 0.11 mg; iodine, 0.06 mg; manganese, 65 mg; magnesium, 0.14 mg; selenium 10 mg; L-Lysine: Monochlorohydrate, 98.5% purity, 78% L-Lysine; DL-Methionine: 98% purity min.

### Data collection and quantification of growth performance, tissue accretion, and gain composition

After the initial 6 days of adaptation, the length of the experimental period ranged from 67 (for the *ad libitum* group) to 135 days (for the LP group), after which animals were slaughtered. *Ad libitum*-fed pigs were tested for a shorter period compared to restricted-fed animals to mimic the scenario under which RFI would be tested in practice, as estimating RFI over extended growth periods, and especially in heavy pigs would pose operational and economic challenges in the testing procedures.

During the experimental period, animals were weighed approximately every 2 weeks. Ultrasonic measurements of BF were collected less frequently than body weight measures, but always in a weighing session, including the first and the last one. Individual BF was measured above the last rib at approximately 5.5–8.0 cm from midline (increasing the distance with increasing BW) [21]. The number of repeated BW and BF observations per pig was on average 8.8 ± 1.54 and 5.1 ± 1.26, respectively.

Individual BW (kg) and BF (mm) measures were used to estimate empty body weight (**EBW**, kg), body lipid mass (**BL**, kg), and body protein mass (**BP**, kg) using equations previously developed for pigs of comparable body weight and age [17,21]. Estimates were obtained as follows:

$$EBW=0.914\times {BW}^{1.008}$$ (1)

$$BL=\frac{9.17+(0.7\times BF)}{100}\times BW$$ (2)

$$BP=0.1353\times {\left(EBW-BL\right)}^{1.1175}$$ (3)

Variation in individual EBW, BL and BP over time was not linear. Six non-linear models, namely Weibull, generalized von Bertalanffy, 2-parameter Beta, 3-parameter Beta, U-Gompertz, and Logistic, were considered to describe the dynamics of EBW, BL and BP over time (Table 2). For each pig, the six models were fitted to all the available records of EBW, BL or BP. To facilitate the estimation of the parameters of the individual curves, one record containing arbitrary values for BW and BF at day 1 (1 kg and 1 mm, respectively), from which arbitrary initial values of EBW, BL and BP were obtained, was added to the set of recorded measures of each animal. The model parameters were estimated using the function *nlsLM* of the *minpack*.*lm* R package [22]. Parameter estimates obtained using different starting values to initialise the model solving iterative process exhibited extremely high correlations (r > 0.99). Also, the mean and variance of the difference between estimates of a given parameter were extremely low indicating low sensitivity of estimates to the starting values attributed to the model parameters. The goodness of fit of each model was assessed using the R^2^ and root mean square error (**RMSE**) which were computed considering only the actual set of recorded measures of each animal. The U-Gompertz non-linear model [23] exhibited the best fitting (Table 2) and was then chosen to model the individual growth curves for EBW, BL, and BP. Mean and SD of parameter estimates and goodness of fit statistics for the U-Gompertz model fitted to the individual data of each pig are reported in S1 Table.

**Table 2:** Models used to describe growth and goodness of fit for body weight.

Model	Function^a^	Reference	Goodness of fit	
			R^2^	RMSE^b^
U-Gompertz	$y_{t}=W_{0}\times exp\left(log(\frac{A}{W_{0}})\times (1-exp(-\frac{e\times K_{U}\times t}{A}))\right)$	[23]	0.9998 ± 0.0002	0.025 ± 0.011
Weibull	$y_{t}=A\times (1-exp(-a\times t^{b}))$	[24]	0.9986 ± 0.0014	2.039 ± 0.942
3 parameter-Beta	$y_{t}=A\times \left(1+\frac{T_{e}-t}{T_{e}-T_{m}}\right)\times {\left(\frac{t}{T_{e}}\right)}^{\frac{T_{e}}{T_{e}-T_{m}}}$	[25]	0.9986 ± 0.0015	2.039 ± 0.956
Generalized von Bertalanffy	$y_{t}=A\times {\left(1-exp(-k\times t)\right)}^{v}$	[26]	0.9985 ± 0.0014	2.059 ± 0.955
Logistic	$y_{t}=\frac{A}{1+exp(-k\times (t-T_{m}))}$	[27]	0.9946 ± 0.0036	4.429 ± 1.246
2 parameter-Beta	$y_{t}=A\times (2T_{e}-t)\times \frac{t}{T_{e}^{2}}$	[25]	0.9330 ± 0.0355	18.574 ± 5.498

^a^ *y*_*t*_ is the observed weight at age *t*; *W*_0_ is the weight at age 0; A is the upper asymptotic value of weight; K_*U*_ is the absolute maximum growth rate (i.e., the growth rate at the inflection point); *a* and b are parameters that determine the shape of the curve; T_*e*_ is the age when the asymptotic value of weight is reached; T_*m*_ is the age at the inflection point where the growth rate is maximized; v controls the asymmetry and the shape of the curve determining the speed with which the animal attains A; k controls the steepness of the curve and is the slope at the inflection point.

^b^ RMSE: Root mean squared error.

For each pig, initial and final EBW, BL and BP in the experimental period were obtained from the equations of the individual U-Gompertz growth curves and used to compute the average daily gain in empty body weight (**EBW**_**ADG**_, kg/day), in body lipid (**BL**_**ADG**_, kg/day) and body protein (**BP**_**ADG**_, kg/day), which were considered measures of tissue accretion, and the empty body weight at the midpoint (**EBW**_**MID**_, kg) of the experimental period. The individual average daily feed intake (**ADFI**, kg/day) was calculated as the total feed intake (kg) recorded by the electronic feeders divided by the days on test. The feed conversion ratio (**FCR**, kg/kg) was computed as the ratio of ADFI to EBW_ADG_. The percentage ratios of BL_ADG_ to EBW_ADG_ (**PL**, %) and of BP_ADG_ to EBW_ADG_ (**PP**, %) were used as measures of composition of weight gain.

### Computation of residual feed intake for *ad libitum*-fed pigs

As non-limiting feed availability is required to estimate RFI, only the data of the pigs fed *ad libitum* were used in the computation of RFI. RFI (kg/day) was computed as the residual term (e_*ij*_) of the following linear model:

$${ADFI}_{ij}=\mu +{sex}_{i}+\beta_{1}{EBW}_{{MID}_{ij}}+\beta_{2}{BL}_{{ADG}_{ij}}+\beta_{3}{BP}_{{ADG}_{ij}}+e_{ij}$$ (4)

where ADFI_*ij*_ was the ADFI of the animal, μ was the overall intercept of the model, sex was the fixed effect of sex (*i*: 1 = gilts, 2 = barrows), and β_1_, β_2_, and β_3_ were the linear regression coefficients of ADFI on EBW_MID_, BL_ADG_ and BP_ADG_, respectively. In the model, EBW_MID_ accounted for variation in ADFI due to average requirements for maintenance whereas BL_ADG_ and BP_ADG_ for the ones due to tissue accretion.

### Sire classification for *ad libitum* residual feed intake

Data of the *ad libitum* group were used to estimate the additive genetic effects on RFI of the 23 sires. The number of *ad libitum*-fed offspring per sire was on average 9 ± 5, and ranged from 3 to 25. The additive genetic effects of the sires on RFI (i.e., breeding values, **EBV**) were estimated using the BLUPf90+ software [28] under the following mixed linear animal model:

$$y_{ij}=\mu +{{batch}_{i}+animal}_{ij}+e_{ij}$$ (5)

where *y* was the individual RFI phenotype, μ was the overall intercept of the model, batch was the random effect of the batch (*i*: 1, …, 4), and animal was the random animal additive genetic effect of the pig (*j*: 1, …, 205). Additive genetic effects, batch effects and residuals of the model were assumed to be *a-priori* normally distributed with zero means and variance equal to $A\sigma_{a}^{2}$, $I_{b}\sigma_{b}^{2}$ and $I_{e}\sigma_{e}^{2}$, respectively, where **A** is the numerator relationship matrix, **I**_**b**_ and **I**_**e**_ are identity matrices of appropriate order and $\sigma_{a}^{2},\sigma_{b}^{2}$, and $\sigma_{e}^{2}$ are known variance components. Additive relationships in **A** were computed using a pedigree file of 1909 animals spanning 22 generations (205 animals with RFI records and 1702 ancestors). Pedigree information was obtained from the historical database of the Goland C21 line.

The sires were ranked based on the solutions of the animal model and allocated to 3 classes: 25% lowest EBV (low-RFI class, **LRFI**, n = 6), 50% medium EBV (medium-RFI class, **MRFI**, n = 11), and 25% highest EBV (high-RFI, **HRFI**, n = 6). Mean ± SD of the sire estimated additive genetic effects on RFI across sire classes are reported in S2 Table.

### Effects of sire classification for *ad libitum* RFI on traits of the restricted-fed progeny

Effects of sire classification for RFI on traits of the restricted-fed progeny were estimated using the joint data of MP and LP groups. Each animal had one observation for each trait, as the measures were the average of the period. The number of restricted-fed pigs per RFI sire class was 68, 81, and 62 for LRFI, MRFI, and HRFI, respectively. The number of restricted-fed offspring per sire was on average 9 ± 5, and ranged from 3 to 23. Each sire class had offspring in both the MP and LP treatment groups: there were 30 LRFI, 45 MRFI, and 29 HRFI pigs in the MP group and 38 LRFI, 36 MRFI, and 33 HRFI pigs in the LP group.

The data were analysed using the following mixed linear model:

$$y_{ijklmn}=\mu +{sex}_{i}+{sire\;class}_{j}+{batch}_{k}+{treatment}_{l}+pen{\left(batch\times treatment\right)}_{m:kl}{+e}_{ijklmn}$$ (6)

where y was an observation on ADFI, FCR, EBW_ADG_, BL_ADG_, BP_ADG_, PL or

PP, μ was the overall intercept of the model, sex was the fixed effect of sex (*i*: 1 = barrow, 2 = gilt), sire class was the fixed effect of the sire class for RFI (*j*: 1 = LRFI, 2 = MRFI, 3 = HRFI), batch was the random effect of the batch (*k*: 1, …, 4), treatment was the fixed effect of the treatment group (*l*: 1 = MP, 2 = LP), and pen (batch × treatment) was the random effect of the pen nested within the batch × treatment interaction. Differences between means of MP and LP groups were tested by F-tests using the pen (batch × treatment) interaction as the error term. Significance of random effects was tested through log-likelihood ratio tests by comparing the likelihood of the full model (which includes the random effect of concern) with that of a reduced model (which excludes the random effect of concern). All analyses were performed using the packages *lmerTest* and *lsmeans* of the R software [22] and differences were considered significant when p < 0.05.

Model 6 was used also to investigate the effects of sire classification for RFI on parameters of the individual growth curves for EBW, BL, and BP. The least square means of the sire class effects on the parameters of individual curves were then used as parameters of the average curve of each sire class. The first derivative of the average curve of each sire class, which describes the evolution over time of the growth rate in EBW, BL or BP (**EBW**_**DG**_, **BL**_**DG**_ and **BP**_**DG**_, respectively), was obtained through symbolic differentiation. Differentiation was performed using the *nlsDeriv* function of the R package *nlsr*.

The difference between curves was inferred using F-tests for W_0_, A and K_U_ parameters. Differences in W_0_ were not relevant as the parameter was heavily influenced by the arbitrary values of EBW, BL and BP set for day 1, which were equal for all animals, and the variability in the estimation of W_0_ depended exclusively on the adaptation of the individual fitted curves to the values that were actually recorded at subsequent ages. For this reason, EBW, BL and BP curves, as well as their first derivatives, were considered to be different across sire classes only in the case of statistically significant differences in A or K_U_.

## Results

### Feed efficiency, growth, and gain composition of *ad libitum*-fed pigs

Mean and SD of the investigated traits for the *ad libitum*-fed pigs are reported in Table 3. At the end of the experimental period, when animals had an age of 221 ± 3 days, the BW was 161.16 ± 15.78 kg, and EBW, BL, and BP were on average 95%, 25%, and 16% of BW, respectively. The average daily gain was 0.89 kg/day. The *ad libitum* group showed a large variability in ADFI (SD = 0.498 kg/day), which ranged from 1.69 to 4.45 kg/day, and in RFI (SD = 0.245 kg/day). The feed conversion ratio was on average 3.6 kg/kg. BL_ADG_ and BP_ADG_ accounted for 36% and 17% of EBW_ADG_, respectively.

**Table 3:** Descriptive statistics of traits measured in *ad libitum*-fed pigs (N = 205).

Trait	Abbreviation	Mean ± SD^a^
At the end of the experimental period		
Body weight (kg)	BW	161.16 ± 15.78
Empty body weight (kg)	EBW	153.41 ± 15.14
Body lipid mass (kg)	BL	40.52 ± 7.98
Body protein mass (kg)	BP	26.54 ± 2.29
EBW at midpoint (kg)	EBW_MID_	125.95 ± 13.10
Average of the experimental period		
Daily gain in EBW (kg/day)	EBW_ADG_	0.892 ± 0.140
Daily gain in BL (kg/day)	BL_ADG_	0.323 ± 0.074
Daily gain in BP (kg/day)	BP_ADG_	0.147 ± 0.021
Daily feed intake (kg/day)	ADFI	3.210 ± 0.498
Feed conversion ratio (kg/kg)	FCR	3.597 ± 0.314
Residual feed intake (kg/day)	RFI	0.000 ± 0.245
BL_ADG_ to EBW_ADG_ ratio (%)	PL	35.83 ± 5.12
BP_ADG_ to EBW_ADG_ ratio (%)	PP	16.59 ± 1.69

^a^ Mean and standard deviation (SD) of the raw data.

### Feed efficiency, growth, and gain composition of restricted-fed pigs

Mean and SD of the investigated traits for the restricted-fed pigs are reported in Table 4. At the end of the experimental period, the animals weighed 166.09 ± 8.83 kg and EBW, BL, and BP averaged 95%, 25%, and 17% of BW, respectively, whereas EBW_ADG_, BL_ADG_ and BP_ADG_ were 0.61, 0.22 and 0.11 kg/day, respectively. As expected, ADFI exhibited a very limited variation (SD = 0.150 kg/day), considerably smaller than that of the *ad libitum*-fed pigs, and ranged from 2.10 to 2.88 kg/day. The low variation in ADFI of restricted-fed pigs led to a low variation also in EBW_ADG_, BL_ADG_ and BP_ADG_. The feed conversion ratio was on average 4.5 kg/kg. BL_ADG_ and BP_ADG_ to EBW_ADG_ ratio (PL and PP) were 37% and 17.5%, respectively.

**Table 4:** Descriptive statistics of traits measured in restricted-fed pigs (N = 211).

Trait	Abbreviation	Mean ± SD^a^
At the end of the experimental period		
Body weight (kg)	BW	166.09 ± 8.83
Empty body weight (kg)	EBW	158.14 ± 8.47
Body lipid mass (kg)	BL	42.10 ± 4.29
Body protein mass (kg)	BP	27.92 ± 1.81
EBW at midpoint (kg)	EBW_MID_	131.85 ± 9.17
Average of the experimental period	
Daily gain in EBW (kg/day)	EBW_ADG_	0.607 ± 0.077
Daily gain in BL (kg/day)	BL_ADG_	0.224 ± 0.038
Daily gain in BP (kg/day)	BP_ADG_	0.106 ± 0.016
Daily feed intake (kg/day)	ADFI	2.663 ± 0.150
Feed conversion ratio (kg/kg)	FCR	4.457 ± 0.585
BL_ADG_ to EBW_ADG_ ratio (%)	PL	36.98 ± 5.38
BP_ADG_ to EBW_ADG_ ratio (%)	PP	17.49 ± 1.38

^a^ Mean and standard deviation (SD) of the raw data.

### Effects of sire classification for RFI on traits of the restricted-fed progeny

**Fig 1:**
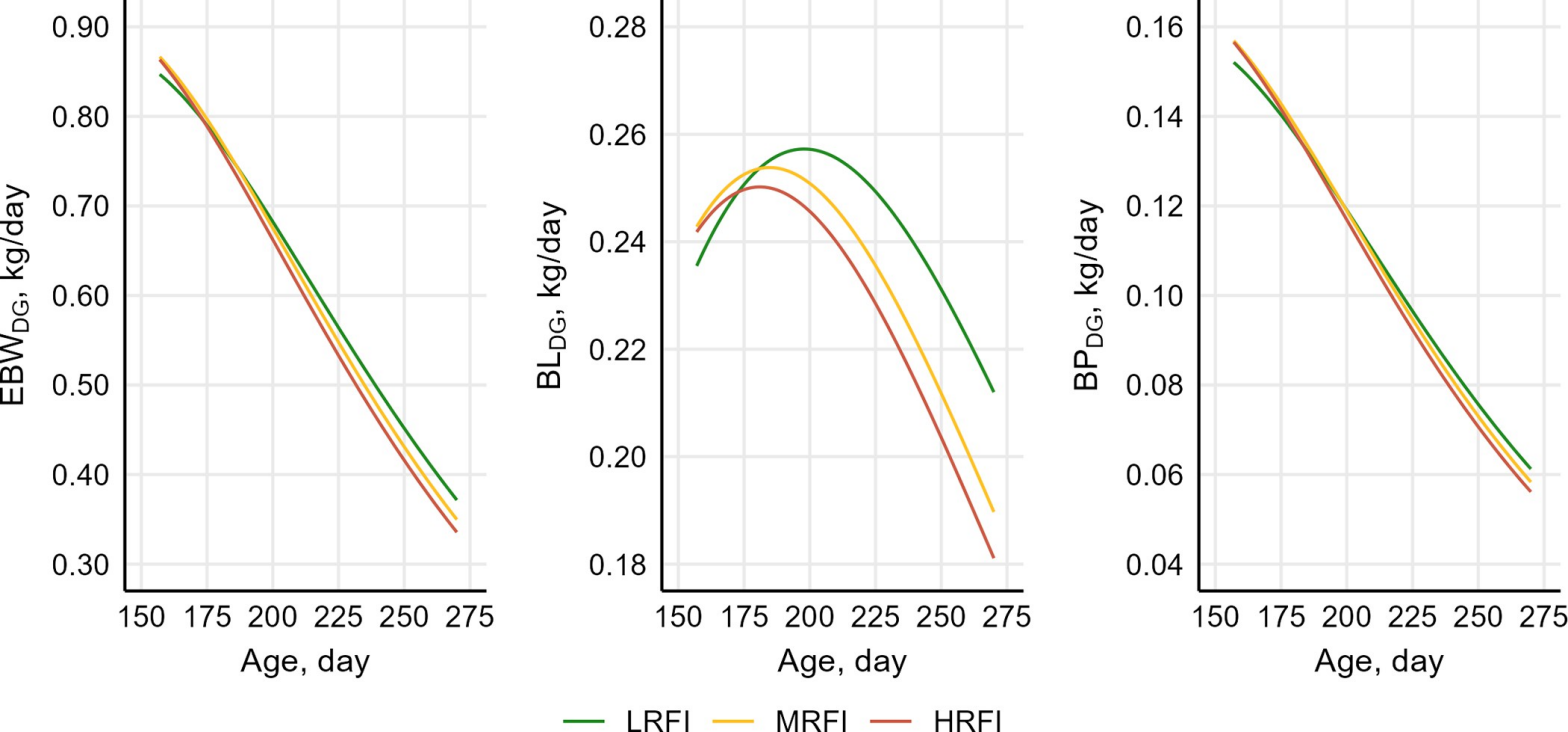
Variation over time in weight gain and tissue accretion of restricted-fed pigs across sire classes for residual feed intake (RFI). Variation over time (age) in growth rate (first derivative of the Gompertz function) of empty body weight (EBW_DG_), body lipids (BL_DG_), and body protein (BP_DG_) of restricted-fed pigs across RFI sire classes: Low RFI (LRFI), medium-RFI (MRFI), and high-RFI (HRFI). Differences between curves were significant for all traits (p < 0.05).

The least square means for the effects of sire classification for RFI on the investigated traits of the restricted-fed progeny are reported in Table 5. Despite the constrained availability of feed and the absence of competition for intake, ADFI was lower for the LRFI group than for MRFI and HRFI pigs (p < 0.001 and p = 0.001, respectively). The daily gain in EBW of the restricted-fed pigs decreased over time, but the rate of decrease tended to be greater (p = 0.059) for the offspring of HRFI and MRFI sires than for the offspring of LRFI sires (Fig 1).

**Table 5:** Effects of sire classification for residual feed intake (RFI) on traits of restricted-fed pigs.

Trait^b^	Class of sire for RFI^a^			LRFI vs MRFI (p-value)	LRFI vs HRFI (p-value)
	HRFI (n = 62)	MRFI (n = 81)	LRFI (n = 68)		
ADFI (kg/day)	2.717 ± 0.018	2.720 ± 0.016	2.633 ± 0.017	< 0.001	0.001
FCR (kg/kg)	4.675 ± 0.150	4.549 ± 0.143	4.156 ± 0.151	< 0.001	< 0.001
EBW_ADG_ (kg/day)	0.605 ± 0.018	0.616 ± 0.018	0.624 ± 0.018	0.464	0.059
BL_ADG_ (kg/day)	0.223 ± 0.011	0.227 ± 0.011	0.230 ± 0.011	0.590	0.252
BP_ADG_ (kg/day)	0.107 ± 0.004	0.108 ± 0.004	0.108 ± 0.004	0.996	0.438
PL (%)	37.09 ± 1.70	37.00 ± 1.67	36.96 ± 1.68	0.959	0.869
PP (%)	17.60 ± 0.28	17.55 ± 0.27	17.33 ± 0.28	0.327	0.242

Least square means (± SE) for the effects of sire class for RFI on feed efficiency, growth performance, tissue accretion, and gain composition traits of the restricted-fed progeny.

^a^ HRFI: High-RFI class; MRFI: Medium-RFI class; LRFI: Low-RFI class.

^b^ ADFI: Average daily feed intake; FCR: Feed conversion ratio; EBW_ADG_: Daily empty body weight gain; BL_ADG_: Daily body lipid gain; BP_ADG_: Daily body protein gain; PL: Percentage ratio of BL_ADG_ to EBW_ADG_; PP: Percentage ratio of BP_ADG_ to EBW_ADG_.

On average, during the experimental period, BP_ADG_ was approximately 100 g/day and was consistent across the three sire groups. Also, the over-time variation in BP_DG_ was similar across sire classes.

The curve for BL_DG_ of MRFI and HRFI pigs differed from that of LRFI pigs. The growth rate of BL in the progeny of MRFI and HRFI sires reached its maximum approximately 2 weeks earlier and decreased at a faster rate than that of the progeny of LRFI pigs (Fig 1). Despite these differences in BL_DG_ curves, sire classes exhibited no significant difference in BL_ADG_ and gain composition (PL and PP). Least square means of the individual U-Gompertz curve parameters are available in S3 Table. As a consequence of lower feed intake, similar or slightly better growth performance, and equal gain composition, FCR of LRFI progeny was 12% lower than that of MRFI and HRFI progeny.

### Effects of the dietary protein content on traits of restricted-fed pigs

**Fig 2:**
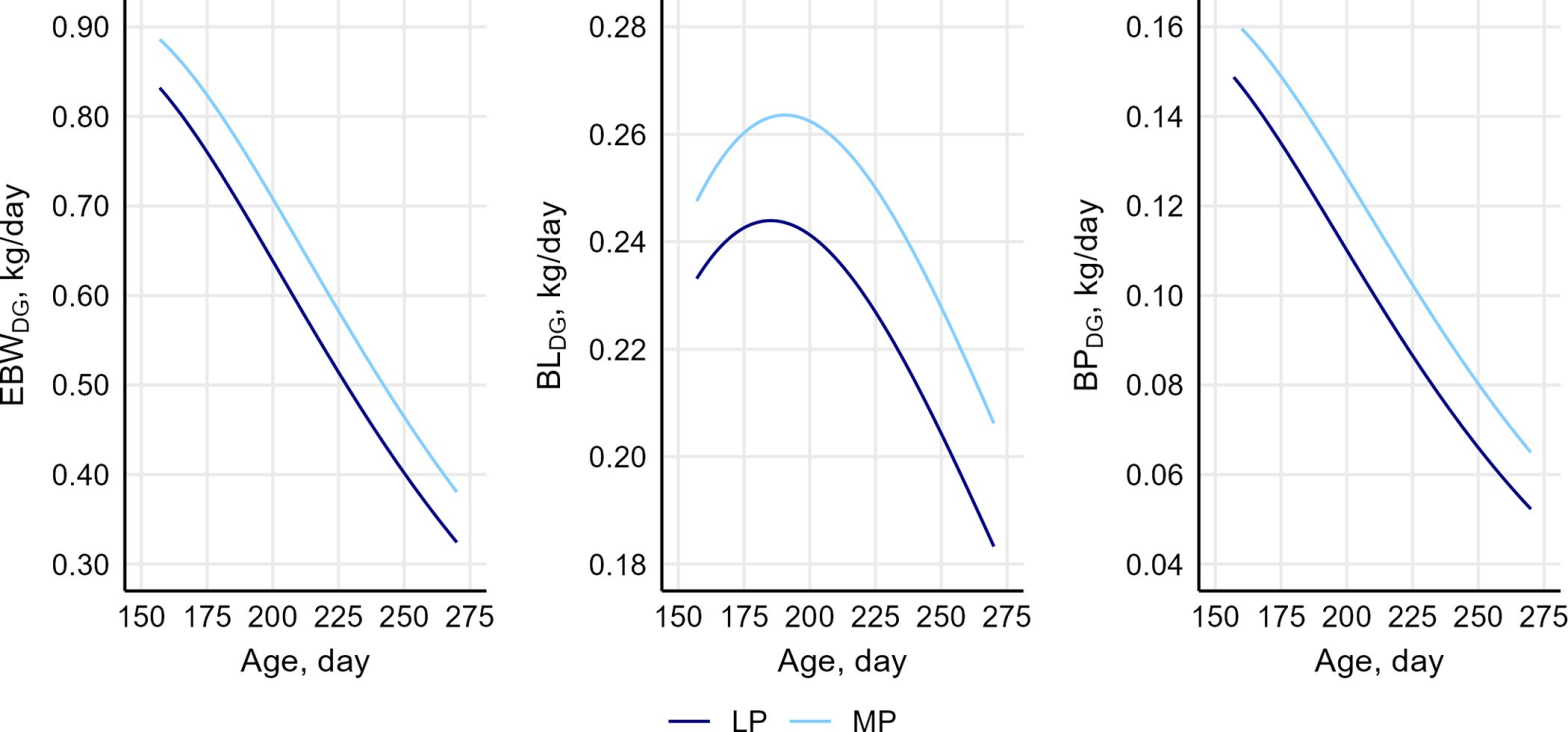
Variation over time in weight gain and tissue accretion of restricted-fed pigs across treatments. Variation over time (age) in growth rate (first derivative of the Gompertz’s function) of empty body weight (EBW_DG_), body lipids (BL_DG_), and body protein (BP_DG_) of restricted-fed pigs across treatment groups: Medium-protein diet (MP) and low-protein diet (LP). Differences between curves were significant for all traits (p < 0.05).

The least square means of the treatment effects (MP and LP) on feed efficiency, growth performance, tissue accretion, and weight gain composition traits of restricted-fed pigs are reported in Table 6. No significant difference in ADFI was observed between MP and LP pigs. As feed intake was similar across the two treatments, feed efficiency, as measured by FCR, was affected by the differences in growth and tissue accretion between the two groups, and was significantly lower (p = 0.007) in MP than in LP. The daily gain in EBW, BL and BP (EBW_DG_, BL_DG_, and BP_DG_) was consistently higher for MP than for LP throughout the experimental period (Fig 2), resulting in high EBW_ADG_ (+16%; p = 0.012), BL_ADG_ (+9%, p = 0.028) and BP_ADG_ (+19%, p = 0.004) for MP relative to LP. Least square means of the individual U-Gompertz curve parameters are reported in S4 Table.

**Table 6:** Effects of the dietary protein treatment on traits of restricted-fed pigs.

Trait^b^	Treatment^a^		p-value
	MP (n = 104)	LP (n = 107)	
ADFI (kg/day)	2.686 ± 0.018	2.693 ± 0.018	0.806
FCR (kg/kg)	4.124 ± 0.139	4.785 ± 0.139	0.007
EBW_ADG_ (kg/day)	0.660 ± 0.005	0.569 ± 0.005	0.012
BL_ADG_ (kg/day)	0.236 ± 0.011	0.217 ± 0.011	0.028
BP_ADG_ (kg/day)	0.117 ± 0.004	0.098 ± 0.004	0.004
PL (%)	35.803 ± 1.652	38.230 ± 1.649	0.030
PP (%)	17.821 ± 0.260	17.169 ± 0.256	0.035

Least square means (± SE) of the effect of dietary protein treatment on feed efficiency, growth performance, tissue accretion, and weight gain composition traits of restricted-fed pigs.

^a^ MP: Medium-protein diet; LP: Low-protein diet.

^b^ ADFI: Average daily feed intake; FCR: Feed conversion ratio; EBW_ADG_: Daily empty body weight gain; BL_ADG_: Daily body lipid gain; BP_ADG_: Daily body protein gain; PL: Percentage ratio of BL_ADG_ to EBW_ADG_; PP: Percentage ratio of BP_ADG_ to EBW_ADG_.

The rate of gain in EBW and BP decreased almost linearly over time. Conversely, BL_DG_ initially increased, reached a maximum at 185 days of age for LP and 191 days for MP, after which it decreased in both treatment groups at an inconsistent rate. As a result of these dynamics, the relative lipid deposition per unit of EBW gain increased over time in both treatment groups, but to a larger extent in LP than in MP. This resulted in a greater PL (38.2 vs 35.8%; p = 0.030) and lower PP (17.2 vs 17.8%; p = 0.035) for LP than MP.

## Discussion

Residual feed intake was estimated from average EBW and tissue accretion (BL and BP) to account for the effects on feed intake due to maintenance requirements, as well as to the different energy expenditure required for the deposition of lean and fat tissue. The models commonly used for RFI computation account for the effects of BW or metabolic BW, daily weight gain, and BF deposition [1,29–31], or incorporate dressing percentage and lean meat content [9]. To the best of our knowledge, this is the first study which considers tissue accretion for the estimation of RFI.

The assessment of RFI started at 96 kg BW, which is in the upper end of the interval used in other studies [2,7,10], and ended at 161 kg BW. Hence, the effects exerted by sire classification on feed efficiency, growth, and gain composition captured by this study refers to a later growth stage, not covered in previous studies. This might in part explain the wide variability in RFI observed in the *ad libitum* pigs, which was remarkably higher than the one observed in previous investigations. In this study, the SD of RFI in the *ad libitum* group was 245 g/day, whereas it ranged from 109 to 158 g/day across four breed groups in an extensive study by [7]. Maintenance needs and growth composition vary across animals characterized by different weights, hence the weight range for measuring RFI is crucial [32]. It is worth mentioning that the pigs involved in this study were generated from nucleus boars of a sire line where selection is addressed to enhance ham quality, but not RFI or other feed efficiency traits. This might also have contributed to the large variability in RFI observed in this study.

### Feed efficiency, growth, tissue accretion, and gain composition of restricted-fed pigs

Besides the different weight range (heavy vs light pigs) of our study in comparison with other literature reports, the different experimental design (sire additive genetic effects vs divergent selection experiments) should also be taken into consideration when comparing results across studies. Even though the study aimed at investigating genetic effects, this was performed by comparing effects of sire classification for RFI on traits of the sire progeny. Conversely, other studies investigated differences between divergently selected lines after multiple generations of selection (from 4 to 9) [2,10].

In the heavy pig production system, energy restriction is used to constrain daily gain in the range 0.65–0.70 kg/day and obtain 170-kg BW pigs at 9 months of age with an adequate carcass and ham fat covering, as required by product specifications [18]. As RFI is the difference between the animal’s observed feed intake and the expected feed intake based on maintenance and growth requirements [5], animals must be able to express their voluntary feed intake and feeding behavior in order to obtain reliable RFI measures. As a consequence of the feeding strategy, the biological variability in feed intake in the restricted-fed group was limited and much lower than that of the *ad libitum* group. For this reason, feed efficiency in restricted-fed pigs cannot be directly measured by RFI.

Compared to the traditional diet (MP), a limiting protein content in the diet (LP diet) leads to lower growth rate, lower protein deposition, and shifts the partition of energy towards fat tissue accretion. This results in an overall lower feed efficiency. Despite the same metabolizable energy intake, the LP pigs utilized 17% less metabolizable energy for protein deposition and 11% more for maintenance than the pigs receiving a MP diet [33].

### Effects of sire classification on traits of the restricted-fed progeny

The progeny of LRFI sires exhibited a 3% lower ADFI (-86 g/day) compared to MRFI and HRFI progeny. Pigs have the ability to adapt their voluntary feed intake based on specific dietary attributes. Pigs fed on diets with reduced energy concentration are prone to maintain constant daily energy intake by eating more feed until feed intake is limited by other factors, such as physical gut capacity or certain dietary components [34]. The reduced ADFI in the LRFI group indicates that the offspring of these sires make a more efficient use of the energy intake. This also implies that genetic differences across sires for *ad libitum* RFI give raise to differences in feed efficiency across sire families even under restricted feeding. Various factors affect variation in RFI, encompassing aspects such as body composition, physical activity, maintenance requirements, digestibility, energetic efficiency, tissue turnover rates, and immune response [1]. Therefore, at the phenotypic level, RFI reflects, in part, the inherent variability in the basal metabolic rate of animals, as supported by results of [35]. It has been hypothesized that a lower number or a reduced activity of mitochondria and lighter visceral weight in pigs selected for low RFI might contribute to explain differences in basal metabolic rate across lines [2]. In addition, pigs selected for low RFI were reported to spend less time standing, leading to reduced physical activity [2]. These results are supported by the findings of [36], indicating a tight association between genetic variation in maintenance energy requirements and genetic variation in RFI. Although the molecular basis of RFI is not fully understood, low RFI pigs have been reported to exhibit lower serum concentration of IGF-1 and leptin, which are known to be associated with cell proliferation and protein turnover rates, and feed intake, respectively [11,37]. This might explain why sire classification, based on the additive genetic effect on *ad libitum* RFI, affects feed efficiency of the restricted-fed progeny.

The lower ADFI exhibited by the LRFI progeny resulted also in a significantly lower FCR, which was affected also by a 3% increase in growth rate of LRFI progeny compared with HRFI. Restricted feeding allows to evaluate how animals utilize a given amount of feed in terms of growth and tissue deposition. Contrarily to what has been reported for *ad libitum*-fed pigs [2,10], when identical amounts of feed were consumed, the most efficient pigs exhibited a slightly faster growth. Our results agree with [13,38]. In those studies, when feed was restricted to 75% or 55% of the *ad libitum* level, pigs belonging to the low RFI line exhibited slightly higher weight gain on an equivalent amount of feed in the weight interval from 25 to 110 kg BW.

In our study, the LRFI progeny consumed less feed, while growing at a similar rate than MRFI or HRFI. This would result in reduced environmental impact and increased profit for farmers. When subject to identical feed restriction, pigs with diminished maintenance requirements or enhanced efficiency in tissue deposition are anticipated to demonstrate either a reduced feed intake and similar growth rate or similar feed intake and faster growth.

No significant differences across sire classes were observed for tissue accretion and gain composition, in contrast with previous studies on restricted-fed pigs [13,38], where the most efficient animals tended to exhibit a higher carcass lean meat content or reduced BF. In this study, the rate of growth and tissue deposition was time- and sire-class-dependent. In the early stages of the experiment, the progeny of LRFI sires tended to show a slightly lower growth and fat deposition compared to the offspring of MRFI and HRFI sires, whereas the opposite was observed during later growth stages.

Offspring of LRFI sires are expected to require, on average, lower energy consumption for maintenance and, at constant feed intake, they can use excess energy for tissue deposition. As partition of energy for protein deposition declines linearly with increasing BW [39], in heavy pigs this results in increased body fat. This indicates that the relationship between feed efficiency traits and carcass composition is time- and feeding regime-dependent. Protein deposition typically reaches a maximum at 60 to 80 kg in growing pigs and decreases thereafter. With aging, an increasing fraction of the available energy is used for lipid deposition [39]. According to [39], a reduction in feed intake, which is associated to low RFI, decreases the energy addressed to fat deposition thus increasing both the proportion of protein deposition and the gain in lean tissue. Moreover, a reduction in feed intake results in a decrease of both protein and lipid deposition in light animals, whereas it mainly affects lipid deposition in heavy animals [39], which can partly explain the discrepancies regarding the relationship between RFI and gain or body fat across studies.

Within the Italian pig production setting, a decreased fat deposition is considered a disadvantage for product quality. The subcutaneous fat thickness of the ham significantly influences salt absorption and water loss during the curing process [16], making it a key factor in determining ham dry-curing aptitude [40]. Based on results of this study, we can hypothesise that feed efficient sires would not affect carcass leanness of heavy pig progeny fed restricted.

## Conclusions

We investigated the effects of sires classified for *ad libitum* residual feed intake on feed efficiency, growth, and gain composition of restricted-fed heavy pigs. Our results indicate that variation in residual feed intake across sire families, assessed under *ad libitum* feeding, is related to across-family differences in feed efficiency detected under restricted feeding. Restricted-fed progeny of efficient sires required less feed while achieving a slight increase in growth rate, with no significant variation in tissue accretion and gain composition compared to progeny of less efficient sires. Hence, albeit limited to a single generation, our results suggest that enhancement of feed efficiency, as measured by RFI, would not decrease fat deposition in heavy pigs fed restricted. This is considered an advantage for the production of traditional Protected Designation of Origin products where fat deposition plays a crucial role. However, considering that variation in RFI impacts tissue metabolism, with potential effects on meat pH, water holding capacity, color, intramuscular fat and post mortem proteolysis, potential effects of variations in RFI on meat quality in heavy pigs should be investigated. In addition, variation in traits genetically correlated with RFI might be detected across multiple generations. This highlights the need to estimate genetic relationships of RFI with growth and body composition to validate the results obtained in the current study.

## Supporting information

S1 Table Estimated parameters and fitting statistics of the U-Gompertz model fitted to individual data. Mean ± SD of the estimated parameters and fitting statistics of the individual U-Gompertz model for the *ad libitum*- and restricted-fed heavy pigs.
(DOCX)

S2 Table Descriptive statistics for the sire estimated additive genetic effects on ad libitum residual feed intake. Mean ± SD of the estimated sire additive genetic effects on *ad libitum* residual feed intake (RFI) across sire classes for RFI.
(DOCX)

S3 Table Effects of the RFI sire classes on the estimated parameters of individual growth curves. Least square means ± SE for the residual feed intake (RFI) sire class effect on the estimated parameters of U-Gompertz model fitted to individual data of restricted-fed pigs.
(DOCX)

S4 Table Effects of the dietary protein treatment on the estimated parameters of individual growth curves. Least square means ± SE for the dietary protein treatment effect on the estimated parameters of U-Gompertz model fitted to individual data of restricted-fed pigs.`
(DOCX)
