# Supplementary material for: Genetic merit of sires for ad libitum residual feed intake affects feed efficiency of restricted-fed heavy pigs but not body weight gain tissue composition
Source: PLoS One. 2024 Oct 17;19(10):e0312307. doi: 10.1371/journal.pone.0312307 (PMC11486364; doi:10.1371/journal.pone.0312307)
Supplement: S1 Table — Mean ± SD of the estimated parameters and fitting statistics of the individual U-Gompertz model for the ad libitum- and restricted-fed heavy pigs. (DOCX) [file pone.0312307.s001.docx]

**S1 Table. Estimated parameters and fitting statistics of the U-Gompertz model fitted to individual data.**

| **Trait^b^** | **Parameter^c^** | **Feeding group^a^** | | |
| --- | --- | --- | --- | --- |
|  |  | ***Ad libitum*** | **Restricted** | |
|  |  |  | **MP** | **LP** |
| EBW | W_0_ (kg) | 0.857 ± 0.005 | 0.854 ± 0.005 | 0.853 ± 0.007 |
|  | A (kg) | 240.59 ± 36.62 | 198.60 ± 12.70 | 183.80 ± 12.70 |
|  | K_U_ (kg/day) | 1.020 ± 0.110 | 0.921 ± 0.072 | 0.883 ± 0.082 |
|  | R^2^ | 0.9998 ± 0.0002 | 0.9997 ± 0.0002 | 0.9997 ± 0.0003 |
|  | RMSE (kg) | 0.023 ± 0.011 | 0.028 ± 0.010 | 0.028 ± 0.011 |
| BL | W_0_ (kg) | 0.089 ± 0.001 | 0.089 ± 0.001 | 0.089 ± 0.001 |
|  | A (kg) | 115.16 ± 52.20 | 71.23 ± 21.59 | 64.90 ± 14.37 |
|  | K_U_ (kg/day) | 0.371 ± 0.116 | 0.263 ± 0.042 | 0.244 ± 0.032 |
|  | R^2^ | 0.9994 ± 0.0006 | 0.9995 ± 0.0004 | 0.9995 ± 0.0005 |
|  | RMSE (kg) | 0.067 ± 0.032 | 0.062 ± 0.027 | 0.058 ± 0.026 |
| BP | W_0_ (kg) | 0.100 ± 0.001 | 0.100 ± 0.001 | 0.100 ± 0.001 |
|  | A (kg) | 38.34 ± 4.55 | 34.76 ± 2.81 | 31.57 ± 2.57 |
|  | K_U_ (kg/day) | 0.179 ± 0.016 | 0.169 ± 0.013 | 0.161 ± 0.015 |
|  | R^2^ | 0.9999 ± 0.0002 | 0.9999 ± 0.0001 | 0.9998 ± 0.0001 |
|  | RMSE (kg) | 0.030 ± 0.016 | 0.031 ± 0.014 | 0.032 ± 0.014 |

Mean ± SD of the estimated parameters and fitting statistics of the individual U-Gompertz model for the *ad libitum*- and restricted-fed heavy pigs.

^a^ MP: medium-protein diet; LP: low-protein diet.

^b^ EBW: empty body weight (kg); BL: body lipid mass (kg); BP: body protein mass (kg).

^c^ W_0_: weight at age 0; A: asymptotic value; K_U_: absolute maximum growth rate at the inflection point; R^2^: coefficient of determination; RMSE: root mean square error.
