# Supplementary material for: Genetic merit of sires for ad libitum residual feed intake affects feed efficiency of restricted-fed heavy pigs but not body weight gain tissue composition
Source: PLoS One. 2024 Oct 17;19(10):e0312307. doi: 10.1371/journal.pone.0312307 (PMC11486364; doi:10.1371/journal.pone.0312307)
Supplement: S2 Table — Mean ± SD of the estimated sire additive genetic effects on ad libitum residual feed intake (RFI) across sire classes for RFI. (DOCX) [file pone.0312307.s002.docx]

**S2 Table.** **Descriptive statistics for the sire estimated additive genetic effects on *ad libitum* residual feed intake.**

| **Sire class for RFI^a^** | **Number of sires** | **Mean ± SD** |
| --- | --- | --- |
| LRFI | 6 | -0.0299 ± 0.0153 |
| MRFI | 11 | 0.0064 ± 0.0155 |
| HRFI | 6 | 0.0378 ± 0.0099 |

Mean ± SD of the estimated sire additive genetic effects on *ad libitum* residual feed intake (RFI) across sire classes for RFI.

^a^HRFI: high-RFI class; MRFI: medium-RFI class; LRFI: low-RFI class.
