# Supplementary material for: Genetic merit of sires for ad libitum residual feed intake affects feed efficiency of restricted-fed heavy pigs but not body weight gain tissue composition
Source: PLoS One. 2024 Oct 17;19(10):e0312307. doi: 10.1371/journal.pone.0312307 (PMC11486364; doi:10.1371/journal.pone.0312307)
Supplement: S3 Table — Least square means ± SE for the residual feed intake (RFI) sire class effect on the estimated parameters of U-Gompertz model fitted to individual data of restricted-fed pigs. (DOCX) [file pone.0312307.s003.docx]

**S3 Table.** **Effects of the RFI sire classes on the estimated parameters of individual growth curves.**

| **Trait^b^** | **Parameter^c^** | **Class of sire for RFI^a^** | | | **Linear contrast**  **(p-value)** | |
| --- | --- | --- | --- | --- | --- | --- |
|  |  | **HRFI**  (n = 62) | **MRFI**  (n = 81) | **LRFI**  (n = 68) | **LRFI vs MRFI** | **LRFI vs HRFI** |
| EBW | W_0_ (kg) | 0.852 ± 0.002 | 0.853 ± 0.002 | 0.856 ± 0.002 | < 0.001 | < 0.001 |
|  | A (kg) | 190.33 ± 3.89 | 192.15 ± 3.83 | 191.24 ± 3.85 | 0.648 | 0.632 |
|  | K_U_ (kg/day) | 0.917 ± 0.026 | 0.911 ± 0.026 | 0.874 ± 0.026 | < 0.001 | < 0.001 |
| BL | W_0_ (kg) | 0.089 ± 0.0003 | 0.089 ± 0.0003 | 0.089 ± 0.0003 | 0.006 | 0.023 |
|  | A (kg) | 65.24 ± 4.98 | 67.36 ± 4.88 | 72.71 ± 4.92 | 0.009 | 0.048 |
|  | K_U_ (kg/day) | 0.250 ± 0.012 | 0.254 ± 0.012 | 0.257 ± 0.012 | 0.226 | 0.530 |
| BP | W_0_ (kg) | 0.100 ± 0.0003 | 0.100 ± 0.0003 | 0.100 ± 0.0003 | < 0.001 | 0.0020 |
|  | A (kg) | 33.21 ± 0.84 | 33.42 ± 0.83 | 32.86 ± 0.83 | 0.418 | 0.164 |
|  | K_U_ (kg/day) | 0.168 ± 0.004 | 0.167 ± 0.004 | 0.158 ± 0.004 | < 0.001 | < 0.001 |

Least square means ± SE for the residual feed intake (RFI) sire class effect on the estimated parameters of U-Gompertz model fitted to individual data of restricted-fed pigs.

^a^ HRFI: high-RFI class; MRFI: medium-RFI class; LRFI: low-RFI class.

^b^ EBW: empty body weight (kg); BL: body lipid mass (kg); BP: body protein mass (kg).

^c^ W_0_: weight at age 0; A: asymptotic value; K_U_: absolute maximum growth rate at the inflection point.
