# Supplementary material for: Genetic merit of sires for ad libitum residual feed intake affects feed efficiency of restricted-fed heavy pigs but not body weight gain tissue composition
Source: PLoS One. 2024 Oct 17;19(10):e0312307. doi: 10.1371/journal.pone.0312307 (PMC11486364; doi:10.1371/journal.pone.0312307)
Supplement: S4 Table — Least square means ± SE for the dietary protein treatment effect on the estimated parameters of U-Gompertz model fitted to individual data of restricted-fed pigs.` (DOCX) [file pone.0312307.s004.docx]

**S4 Table.** **Effects of the dietary protein treatment on the estimated parameters of individual growth curves.**

| **Trait^b^** | **Parameter^c^** | **Treatment^a^** | | **Linear contrast**  **(p-value)** |  |
| --- | --- | --- | --- | --- | --- |
|  |  | **MP**  (n = 104) | **LP**  (n = 107) | **MP vs LP** | |
| EBW | W_0_ (kg) | 0.854 ± 0.002 | 0.853 ± 0.002 | 0.321 | |
|  | A (kg) | 198.54 ± 3.77 | 183.95 ± 3.76 | 0.002 | |
|  | K_U_ (kg/day) | 0.919 ± 0.026 | 0.884 ± 0.026 | 0.021 | |
| BL | W_0_ (kg) | 0.089 ± 0.0003 | 0.089 ± 0.0003 | 0.774 | |
|  | A (kg) | 71.80 ± 4.78 | 65.08 ± 4.76 | 0.054 | |
|  | K_U_ (kg/day) | 02.64 ± 0.012 | 0.244 ± 0.011 | 0.022 | |
| BP | W_0_ (kg) | 0.100 ± 0.0003 | 0.100 ± 0.0003 | 0.096 | |
|  | A (kg) | 34.74 ± 0.81 | 31.59 ± 0.81 | 0.002 | |
|  | K_U_ (kg/day) | 0.168 ± 0.004 | 0.161 ± 0.004 | 0.024 | |

Least square means ± SE for the dietary protein treatment effect on the estimated parameters of U-Gompertz model fitted to individual data of restricted-fed pigs.

^a^ MP: medium-protein diet; LP: low-protein diet.

^b^ EBW: empty body weight (kg); BL: body lipid mass (kg); BP: body protein mass (kg).

^c^ W_0_: weight at age 0; A: asymptotic value; K_U_: absolute maximum growth rate at the inflection point.
